# Coping strategies of Health Care Workers during third wave of Covid

**DOI:** 10.1192/j.eurpsy.2023.887

**Published:** 2023-07-19

**Authors:** M. Theodoratou, P. Kanellopoulou, N. Nikitidis, I. Farmakopoulou

**Affiliations:** 1 Social Sciences, Hellenic Open University, Patras, Greece; 2 Health Sciences, Neapolis University of Pafos, Pafos, Cyprus; 3 Humanistic Sciences, University of Patras, Patras, Greece

## Abstract

**Introduction: Background:**

During the pandemic, health professionals had the unprecedented experience of dealing with a newdisease with high contagiousness and mortality. The workload, but also the fear of disease management, caused significant levels of stress. Each employee managed stress in his own way. This study detects the level of stress experienced by health workers during the pandemic and the coping strategies they selected accordingly.

**Objectives:**

The aim of this study was to investigate the coping strategies used by health professionals during the pandemic of coronavirus.

**Methods:**

The sample comprised 180 health professionals that were working in a public hospital. The data collection tool was an anonymous questionnaire consisting of socio-demographic questions, the Toulouse Scale for coping , and a sub questionnaire to explore health professionals’ views on the pandemic.

**Results:**

Women (73.9%), young individuals (50.6% are up to 35 years old) and TEI graduates (53.9%) predominate in the sample. The sample consisted mainly of nurses (68.3%) and the great majority were contract workers (67%).

The most frequently used dimensions were “Active focus” (Average 3.91/5.00), “Acceptance” (3.86), “Cognitive Control and Planning” (3.61) and “Social Information Support” (3.60).

Also, health workers used more often the strategies of “Social support” (3.45), “Control” (3.33) and “Focus” (3.23), while they use the “Withdrawal” strategy less often (2.25). Finally, respondents used “Positive strategies” more often (3.54) than negative ones.

**Conclusions:**

The health professionals in the present study preferred to ask for information and use cognitive and informative strategies more often and, to a lesser extent, they were overwhelmed by their emotions. The strategies of social support, control and focus were used more often, while the strategy of withdrawal was selected less often. Furthermore, positive strategies were used to a greater extent.

**Disclosure of Interest:**

None Declared

